# Long-term results for pit-picking and flap procedures in primary pilonidal sinus disease

**DOI:** 10.1186/s12893-023-02014-6

**Published:** 2023-04-28

**Authors:** K. Koskinen, J. Harju, K. Hermunen

**Affiliations:** 1 Faculty of Medicine, University of Helsinki, Helsinki, Finland; 2 Helsinki University Hospital, Abdominal Center, Jorvi Hospital, Turuntie 150, PO Box 800, HUS 00029 Espoo, Finland

**Keywords:** Long-term results, Long-term follow-up, Pilonidal disease, Pilonidal sinus disease, Sinus pilonidalis, Bascom, Karydakis, Pit-picking

## Abstract

**Background:**

Pilonidal sinus disease (PSD), a common inflammatory condition of the natal cleft causing morbidity especially in young adults, is a heterogeneous disease group with no consensus regarding its best treatment. Our aim was to report long-term results for primary PSD surgery.

**Methods:**

We retrospectively studied the medical records of 146 patients who underwent primary PSD surgery between November 2010 and October 2015. Of these, 113 underwent either the mini-invasive pit-picking surgery (PSS) (*n* = 55) or asymmetrical excision with local flap (AELF) (*n* = 58); we focused on the outcomes of these two subgroups.

**Results:**

PSD patients who underwent mini-invasive PPS more often succeeded with day surgery (94.5% vs 32.8%, *p* < 0.001), had fewer postoperative complications (9.4% vs 36.2%, *p* = 0.002), and had shorter sick leave (median 14 days vs 21 days, *p* < 0.001) than did AELF patients. Nevertheless, at the first postoperative follow-up visit, both surgery methods healed similarly (75.0% vs 76.8%, *p* = 0.83). Our long-term follow-up, at a median of 9.3 years (range 5.4–10.6), revealed, however, that recurrence after PPS was markedly higher than after AELF (50.9% vs 10.3%, HR 6.65, *p* < 0.001).

**Conclusions:**

PPS, which is a mini-invasive surgical technique often performed under local anaesthesia, is suitable for primary PSD, despite the high recurrence rate in our study, bearing in mind that patient selection is an important factor to consider. Primary PSD with simple sinus formations may benefit from PPS. On the other hand, primary PSD with complex sinus formations may benefit from AELF regardless of the initial slow recovery in our study. Because PSD is a very heterogenous disease, and patients have different risk factors, it is mandatory for the surgeon to master several different surgical techniques. A classification system to aid the surgeon in selecting the right surgical technique for each patient is warranted.

## Introduction

Pilonidal sinus disease (PSD) most commonly arises in the hair follicles of the natal cleft of the sacrococcygeal area, and it predominantly affects young adults [1]. The incidence has been increasing over the past decades. The mean rate of inpatient episodes of PSD per 100,000 male patients increased from 43 in 2005 to 56 in 2017 and in female patients, the mean rate of inpatient episodes per 100,000 rose from 14 in 2005 to 18 in 2017 [2]. Female patients represent approximately 20% of all PSD patients, with the ratio between males and females remaining constant over time [3].

Although PSD is a benign disease, it can cause pain and discomfort, leading to absence from work and school, and can reduce quality of life. Moreover, postoperative complications such as infection and chronic nonhealing wounds are common, and recurrence is high [4]. One systematic review reported an infection rate of 10.4% after midline closure and 6.3% after off-midline closure [1]. In another meta-analysis, five-year recurrence rates ranged from 10.2% to 21.9% depending on the surgical technique [5].

No consensus exists regarding the best PSD-treatment modality [6]. PSD is a very heterogeneous disease group consisting of primary and recurrent disease, acute and chronic manifestations as well as simple and complex sinus formations; this limits the applicability of any single treatment approach [7].

A variety of differing surgical techniques [8] include excision of all involved skin and subcutaneous tissue, followed by either wound closure or open-wound treatment [9], the latter possibly combined with negative-pressure wound therapy (NPWT) to accelerate wound healing [10]. Compared to excisions, several flap-reconstruction techniques such as Karydakis plasty [11], Bascom cleft lift [12], V–Y flap [13] and Rhomboid flaps [14, 15] have, over time, improved the results. Although recovery after flap reconstructions is reportedly good, they are associated with longer hospital stays and longer recovery times compared to mini-invasive treatment options when patients can be discharged on the same day of the surgery.

In recent decades, unsatisfactory results from all of these surgical methods have led to development of minimally invasive PSD treatment [16]. The first such treatment was described by Lord and Millar in 1965 [17]. In 1980, Bascom [18] introduced a similar”pit-picking” technique. Other minimally invasive techniques include phenol treatment [19], fibrin-glue treatment [20], radial laser-probe surgery [21], and endoscopic treatment [22], not to forget the Gips technique, a minimally invasive procedure using trephines [23]. Minimally invasive techniques are generally considered to be associated with shorter hospital stays, reduced postoperative morbidity, and quicker return to normal daily activities when compared to results from traditional excisions and flap-reconstruction techniques [24].

Follow-up times after these differing surgical techniques are relatively short, making evaluation of long-term healing rates and recurrences difficult. One meta-analysis comprising 15 studies from 1995 to 2015 assessed long-term results (mean follow-up time 58–240 months) of PSD surgery, and revealed a rate of relapsing disease at a weighted mean incidence of 13.8% [25]. That meta-analysis included a wide variety of surgical techniques, and when these were assembled into larger groups, showed an incidence of recurrence as follows: 17.9% after open-wound surgery, 16.8% after midline closure, and 10% after off-midline closure. Brusciano et al. [26] reported a recurrence rate of 8.9% after an asymmetric excision with primary closure for primary and recurrent PSD; their median follow-up was 11 years (range 3–22 years).

Recurrences of PSD following surgical treatment may occur up to 20 years later or even longer, with 75% of all recurrences occurring within the first 5 years [27]. Minimally invasive techniques seem most effective, but their relatively short history means that additional long-term follow-ups are vital. To the best of our knowledge, the pit-picking technique’s long-term results have been evaluated only once, after a mean follow-up period of 3.5 years, and there they showed a recurrence rate of 15% [28]. According to Milone et al., long-term follow-up for PSD surgery of at least 5 years should be considered the gold standard [25].

The aim of this study was to retrospectively report long-term follow-up results for primary PSD surgery. Our purpose was both to map patient-specific risk factors and to compare the outcomes of pit-picking surgery (PPS) and asymmetrical excision with local flap (AELF).

## Materials and methods

This retrospective study comprising consecutive patients was conducted in accordance with the Declaration of Helsinki; its authorization number is HUS/155/2021, 16 June 2021.

### Patients

**Fig. 1:**
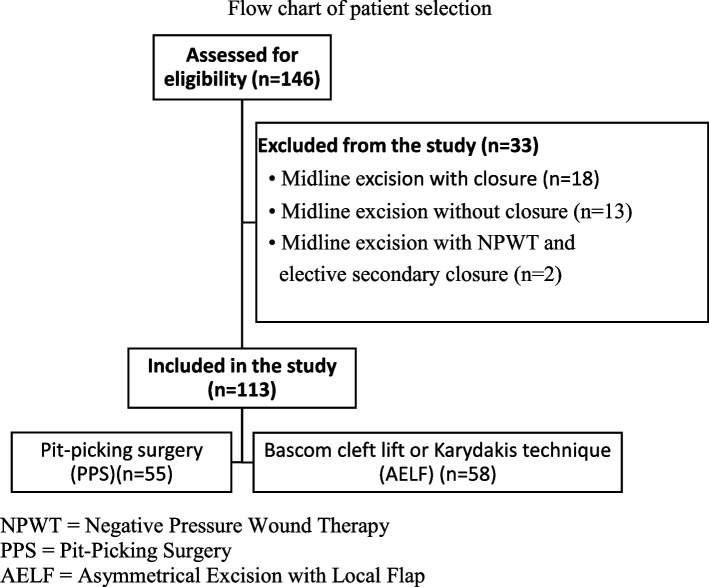
Flow chart of patient selection. NPWT = Negative Pressure Wound Therapy. PPS = Pit-Picking Surgery. AELF = Asymmetrical Excision with Local Flap

Medical records were available for review for 146 consecutive patients who underwent primary PSD surgery for primary chronic PSD at Helsinki University Hospital, Finland, between November 2010 and October 2015. The cut-off date for follow-up was May 17, 2021. We excluded everyone other than those who had been operated on with the pit-picking technique, Bascom cleft lift technique, or Karydakis technique; this provided us with a total of 113 patients (Fig. 1).

We reviewed medical records covering a median of 9.3 years (range 5.4–10.6) postoperatively. All patients underwent surgery performed by eight consultant-level surgeons (senior surgeons) and three residents (doctors in training to become surgeons, that is, junior surgeons), each of whom was free to choose the most suitable surgical technique for each patient. Each patient made at least one postoperative follow-up visit one or two weeks postoperatively; additional visits took place in the event of wound nonhealing (median 2 weeks, range 1–55 weeks).

### Surgical technique

All procedures were performed in the prone position, with a single dose of 1.5 g of cefuroxime and 0.5 g of metronidazole administered intravenously 30 min preoperatively.

PPS is a minimally invasive technique [18] in which the surgeon excises midline pits with a punch, cleans the sinus tracts, and drains the abscess cavity through a lateral incision subsequently left open to heal by secondary intention. Finally, both midline pits and the tract between pits and cavity are closed with a nonabsorbable monofilament suture.

The patients’ medical records sometimes failed to state clearly whether the operation was by the Bascom cleft lift technique or the Karydakis technique; hence we formed the AELF) group by combining the Bascom cleft lift and Karydakis techniques. The Bascom cleft lift technique, also known as the Bascom II or Bascom flap, is an asymmetrical elliptic excision in which the sinus tracts are removed, with excision to the subcutaneous fatty tissue [12]. In the Karydakis flap technique, the sinus tracts are removed down to the sacrococcygeal fascia by an asymmetrical elliptic excision [11]. A flap is made on the contralateral side with cutaneous-subcutaneous fatty tissue, and this tissue is fixed on the defect side, transposing the natal cleft laterally. Finally, the subcutaneous layer is closed with a resorbable polyfilament suture, and the cutaneous layer is closed with a nonabsorbable monofilament suture.

### Anaesthesia

The procedures were performed under local anaesthesia, total intravenous anaesthesia, or spinal anaesthesia. Regardless of the type of anaesthesia, a local anaesthetic (ropivacaine and/or lidocaine adrenalin) was infiltrated into the operating area. The decision as to which type of anaesthesia to use was made together by the surgeon, the patient, and the anaesthesiologist.

### Statistical analysis

Primary endpoints of this study are recurrence of PSD and postoperative complications. Recurrence-free survival (RFS) was defined as the time interval from surgery to recurrence and those patients without recurrence were censored at the date of last follow-up. The surgical technique (PPS vs. AELF) and the patient and surgery characteristics were analysed by univariable Cox proportional hazard models to find factors related to RFS. The results are given as hazard ratios, HR (95% CI) (Table 2). Kaplan–Meier survival analysis and log-rank test were used to compare the difference in RFS between PPS and AELF and to estimate the RFS (%) at 2, 5, and 10 years after surgery. The follow-up time was calculated from surgery to the cut-off date (May 17, 2021). No cases were lost to follow-up before the cut-off date.

The surgical technique (PPS vs. AELF) and the patient and surgery characteristics were analysed by univariable logistic regression models to find factors related to postoperative complications. The results are given as odds ratios, OR (95% CI) (Table 3).

The surgical technique was the only significant predictor for the primary endpoints, so multivariable models were not estimated.

Secondary outcomes were length of sick leave, success of day surgery, and wound healing. Continuous variables are presented as median (range) and categorical variables as frequency (%) and were analysed by the Mann–Whitney U-test and the Pearson chi-squared test, as appropriate.

Analyses were performed with IBM SPSS Statistics for Windows (version 28.0, Armonk, NY, USA, IBM Corp.). A two-sided p-value < 0.05 was considered statistically significant.

## Results

**Fig. 2:**
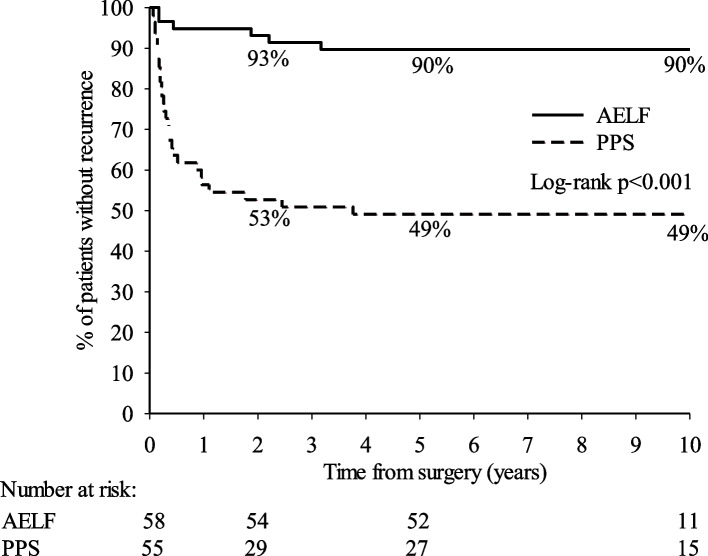
Kaplan–Meier curves showing recurrence-free survival after primary pilonidal disease surgery stratified by surgical technique: Pit-picking surgery (PPS) or Asymmetrical excision with local flap (AELF). Percentages indicate the percentage of patients without recurrence at 2, 5 and 10 years after surgery

We included 113 patients in this retrospective study, which showed a male predominance (77.0%) and a median age of 26 (Table 1). The data collected comprised patient characteristics, the surgical technique chosen, spillage of pus from the sinus tract during surgery, and number of sinus pits (Table 1), as well as recurrence and recurrence time (Fig. 2 and Table 2), postoperative complications (Table 3), length of sick leave, success of day surgery, and healing at follow-up (Table 4). Analysis of the results at a median of 9.3 years revealed 34 recurrences (30.1%), these occurring at a median of 4 months postoperatively (range 3 weeks to 45 months).

**Table 1:** Patient and surgery characteristics of patients undergoing primary pilonidal sinus disease (PSD) surgery; Pit-picking surgery (PPS) or Asymmetrical excision with local flap (AELF)

	All patients(*n* = 113)	PPS (*n* = 55)	AELF (*n* = 58)	*p*-value ^a^
	n (%)	n (%)	n (%)	
*Patient characteristics*				
Age, years				0.03
Median (range)	26 (16–66)	24 (16–59)	28 (16–66)	
BMI, kg/m^2^				0.29
Median (range)	28.4 (17.9–44.5)	28.3 (17.9–44.5)	29.1 (21.6–44.1)	
Missing values	56	24	32	
Gender				
Male	87 (77.0)	42 (76.4)	45 (77.6)	0.88
Female	26 (23.0)	13 (23.6)	13 (22.4)	
Diabetes				
Yes	7 (6.2)	2 (3.6)	5 (8.6)	0.27
No	106 (93.8)	53 (96.4)	53 (91.4)	
Smoking				
Yes	46 (68.7)	20 (60.6)	26 (76.5)	0.16
No	21 (31.3)	13 (39.4)	8 (23.5)	
Missing values	46	22	24	
Previous abscess incision				
Yes	61 (54.5)	30 (54.5)	31 (54.4)	0.99
No	51 (45.5)	25 (45.5)	26 (45.6)	
Missing values	1	0	1	
*Surgery characteristics*				
Spillage of pus during surgery				
Yes	10 (8.8)	2 (3.6)	8 (13.8)	0.06
No	103 (91.2)	53 (96.4)	50 (86.2)	
Sinus number				
< 2	20 (21.3)	13 (24.1)	7 (17.5)	0.44
≥ 2	74 (78.7)	41 (75.9)	33 (82.5)	
Missing values	19	1	18	

^a^ Mann–Whitney U test for continuous variables and Pearson’s chi-square test was used for categorical variables

**Table 2:** Patient and pilonidal surgery characteristics to predict recurrence-free survival

Predictors	N	HR^a^	95% CI	*p*-value
Surgery^b^				
AELF	58	Reference		
PPS	55	6.65	2.75–16.10	< 0.001
Gender				
male	87	Reference		
female	26	1.14	0.53–2.44	0.74
Age (years)				
< 26	53	Reference		
≥ 26	60	0.55	0.28–1.08	0.08
BMI (kg/m^2^)				
< 25	13	Reference		
≥ 25	44	3.22	0.75–13.82	0.12
Diabetes				
No	106	Reference		
Yes	7	0.88	0.21–3.66	0.86
Smoking				
No	21	Reference		
Yes	46	0.78	0.33–1.83	0.57
Previous incision of abscess				
No	51	Reference		
Yes	61	1.05	0.53–2.07	0.89
Spillage of pus during surgery				
No	103	Reference		
Yes	10	0.61	0.15–2.53	0.49
Sinus number				
< 2	20	Reference		
≥ 2	74	1.25	0.52–3.05	0.62

^a^ *HR* hazard ratios were calculated by univariable Cox proportional hazards models

^b^ *PPS* Pit-picking surgery, *AELF* Asymmetrical excision with local flap

**Table 3:** Patient and pilonidal surgery characteristics to predict postoperative complications

Predictors	patients with postoperative complications				
	N	(%)	OR^a^	95% CI	*p*-value
Surgery^b^					
AELF	21/58	(36.2)	0.18	0.06–0.53	0.002
PPS	5/53	(9.4)			
Gender					
male	20/85	(23.5)	0.98	0.34–2.76	0.96
female	6/26	(23.1)			
Age (years)					
< 26	12/52	(23.1)	1.04	0.43–2.50	0.94
≥ 26	14/59	(23.7)			
BMI (kg/m^2^)					
< 25	0/11^c^	(0.0)	4.61^c^	0.54–39.49	0.16
≥ 25	13/44	(29.5)			
Diabetes					
No	22/104	(21.2)	4.97	1.03–23.87	0.05
Yes	4/7	(57.1)			
Smoking					
No	2/19	(10.5)	3.72	0.76–18.31	0.11
Yes	14/46	(30.4)			
Previous incision of abscess					
No	15/50	(30.0)	0.52	0.21–1.28	0.15
Yes	11/60	(18.3)			
Spillage of pus during surgery					
No	25/101	(24.8)	0.34	0.04–2.80	0.31
Yes	1/10	(10.0)			
Sinus number					
< 2	5/19	(26.3)	0.79	0.25–2.51	0.68
≥ 2	16/73	(21.9)			

^a^ *OR* Odds ratios were calculated by univariable logistic regression models

^b^ *PPS* Pit-picking surgery, *AELF* Asymmetrical excision with local flap

^c^ 0/11 (0.0) was replaced by 1/11 (0.09) before analysis

**Table 4:** Postoperative results of patients undergoing primary pilonidal sinus disease (PSD) surgery Pit-picking surgery (PPS) or Asymmetrical excision with local flap (AELF)

	All patients (*n* = 113)	PPS (*n* = 55)	AELF (*n* = 58)	*p*-value ^a^
Length of sick leave, days				< 0.001
Median (range)	14 (7–161)	14 (7–114)	21 (13–161)	
Missing values	33	9	24	
Success of day surgery n (%)	71/113 (62.8)	52/55 (94.5)	19/58 (32.8)	< 0.001
Healed wound at the follow-up visit n (%)	82/108 (75.9)	39/52 (75.0)	43/56 (76.8)	0.83

^a^ Mann–Whitney U test and Pearson’s chi-squared test

The AELF patients were older (*p* = 0.03) than the PPS patients. In regards to body mass index (BMI), gender, diabetes, smoking habits, and previous abscess incision, no statistical difference emerged between these two groups. The AELF produced greater spillage of pus and more often had more than one sinus compared to the PPS, but these differences were not statistically significant (Table 1).

Figure 2 shows the recurrence-free survival for our two PSD surgery groups: PPS and AELF at 2, 5, and 10 years after surgery. For PPS, 53% of the patients were without a recurrence at 2 years, and 49% of the patients were without a recurrence at 5 and 10 years. For AELF, 93% of the patients were without a recurrence at 2 years, and 90% of the patients were without a recurrence at 5 and 10 years. Thus the PPS patients had a 6.65-fold higher risk of recurrence compared to that of the AELF patients. Besides the surgical technique, we found no other statistically significant risk factors for recurrence (Table 2).

Postoperative complications afflicted 5 patients (9.4%) in the PPS group, these including 2 infections and 3 bleedings or haematomas, and 21 patients (36.2%) in the AELF group, these including 12 infections, 5 bleedings or hematomas, and 4 seromas. Thus the PPS patients had fewer postoperative complications than did the AELF patients (*p* < 0.002). Overweight or obese patients, as well as diabetic and smoking patients experienced more postoperative complications, but besides the surgical technique, we found no statistically significant risk factors for complications (Table 3).

The PPS led to a statistically significantly higher success rate for day surgery (*p* < 0.001) and a shorter length of sick leave (*p* < 0.001) than for the AELF group. Healing rates at the postoperative follow-up visit showed no difference between these two groups (*p* = 0.83), but in long-term follow-up, PPS patients had a higher recurrence rate than did the AELF patients (*p* < 0.001) (Table 4).

## Discussion

Our long-term study revealed a statistically significant difference regarding the recurrence rate between PPS and AELF. For PPS, 49% of the patients were without a recurrence at 10 years, whereas the corresponding figure for AELF was 90%. The recurrences appeared, in both groups, within 4 years of follow-up. PPS patients had a higher risk of recurrence compared to that of AELF patients, but in regards to postoperative recovery, the figures were quite the opposite. Patients undergoing PPS had a higher success rate of day surgery and required a shorter sick leave for recovery. Nevertheless, at the postoperative follow-up visit, both procedures showed similar healing rates.

In the surgical literature, when compared to more traditional techniques, minimally invasive techniques are in general associated with better postoperative recovery and with fewer postoperative complications [24].

Follow-up times among PSD studies are, however, often short, and we could only find a few studies focused on surgical techniques similar to those of our study which had a follow-up time of more than five years [26, 29, 30]. The Bascom II technique, after a five-year-long follow-up, had a 23.8% recurrence rate [29]. The Karydakis technique had a recurrence rate of 8.8% after a median follow-up of 11 years in one study [30], and of 11.0% after a median follow-up of 33 months in another [31]. Brusciano et al. reported an 8.9% recurrence rate with a median follow-up of 11 years after D-shape asymmetric excision [26], a surgical technique somewhat similar to our AELF technique. The recurrence rates in these studies correspond well to our own recurrence rate after long-term follow-up of AELF surgery, which was 10.3%.

For assessing and comparing surgical techniques and risk factors in regards to short-term and long-term outcomes, long-term follow-up studies are of course important. One particular surgical technique may provide excellent short-term outcomes, but less favorable long-term outcomes and vice versa. For instance, short-term follow-up revealed that our PPS patients recovered well, the success rate of day surgery was high (94.5%), and the length of sick leave was moderate (14 days), whereas the long-term follow-up revealed a disappointingly high recurrence rate (50.9%).

Bascom et al. reported a recurrence rate of 15% at 3.5 years which differs significantly from our finding of 50.9% at 10 years [28].

One explanation for the high long-term recurrence rate in PPS may be the mini-invasive nature of the operation itself. In PPS, larger sinus tracts are cleaned, and only partially removed, whereas smaller tracts may go unrecognised and be left uncleaned, leaving remnants in the skin and in the subcutaneous adipose tissue which can act as a possible source of recurrent disease. Another explanation could be our follow-up time of nearly 10 years. Recurrences can occur up to 20 years after surgery, although 75% occur within 5 years [27].

Known risk factors for complications in PSD surgery are obesity [32], diabetes [33], smoking [32], plus spillage of pus during surgery [21], and sinus number [34]. In our study, however, none of these: obesity, smoking, spillage of pus during surgery, or sinus number as risk factors reached statistical significance. The high number of missing values regarding the patients' BMI, smoking habits, and sinus numbers could explain this lack of statistical significance. As for spillage of pus during surgery, that the total number of patients was only 113 could explain why this risk factor did not reach statistical significance.

The retrospective nature of this study is a limitation, as are the missing numbers for some patient-specific risk factors as well as the rather small subgroups. Furthermore, we lacked clear data on the exact techniques used in the AELF group.

The long follow-up time of primary PSD surgery is definitely a strength, as is our presentation of two different surgical techniques. Long-term follow-ups of PSD surgery are rare and most only report on one technique. Moreover, to reduce population heterogeneity, we covered only primary PSD surgery. Other strengths are the Finnish health care system’s electronic medical records, providing consistent patient record data from both specialized hospital care and primary health care, This makes retrospective follow-ups feasible. None of the study patients moved to other regions during the follow-up period, thus enabling reliable follow-up data.

## Conclusion

PPS, being a mini-invasive surgical technique often done in local anaesthesia, makes it suitable for primary PSD, despite the high recurrence rate in our study, bearing in mind that patient selection is an important factor to consider. Primary PSD with simple sinus formations may benefit from PPS, whereas primary PSD with complex sinus formations probably would not benefit from PPS.

AELF is a rather invasive surgical technique for primary PSD compared to PPS and in our study it associated with initial slow recovery. Therefore we would not recommend AELF for the treatment of primary PSD with simple sinus formations. However, we would recommend AELF for primary PSD with complex sinus formations.

Because PSD is a very heterogenous disease, and patients have different risk factors, it is impossible to manage primary and recurrent disease, acute and chronic manifestations as well as simple and complex sinus formations with one surgical technique. It is therefore mandatory for the surgeon to master several different surgical techniques. A classification system to aid the surgeon in selecting the right surgical technique for each patient is warranted.

## Data Availability

Available upon request.

## Abbreviations

PSD: Pilonidal sinus disease
PPS: Pit-picking surgery
AELF: Asymmetrical excision with local flap
NPWT: Negative-pressure wound therapy
